# Need of guidance in disabling and chronic migraine identification in the primary care setting, results from the european MyLife anamnesis survey

**DOI:** 10.1186/s12875-021-01402-2

**Published:** 2021-03-20

**Authors:** Angel L. Guerrero, Andrea Negro, Philippe Ryvlin, Kirill Skorobogatykh, Rainel Sanchez-De La Rosa, Heike Israel-Willner, Christina Sundal, E. Anne MacGregor

**Affiliations:** 1 Neurology Department, Headache Unit, Hospital ClínicoUniversitario, Valladolid, Spain; 2 Department of Medicine, University of Valladolid, Valladolid, Spain; 3 Institute for Biomedical Research of Salamanca (IBSAL), Salamanca, Spain; 4 Regional Referral Headache Centre, Sant’Andrea Hospital, Rome, Italy; 5 Department of Clinical and Molecular Medicine, Sapienza University, Rome, Italy; 6 Department of Clinical Neurosciences, CHUV, Lausanne, Switzerland; 7 University Headache Clinic, Moscow, Russia; 8 Region Europe Medical Department, Novartis Pharma AG, Basel, Switzerland; 9 Specialized Center of Neurology Berlin (NFZB), Berlin, Germany; 10 Department of Neurology, Neuroclinic Norway, Lillestrøm, Norway; 11 Department of Clinical Neuroscience, Institute of Neuroscience and Physiology, The Sahlgrenska Academy, University of Gothenburg, Gothenburg, Sweden; 12 Barts Health NHS Trust, London, UK; 13 Centre for Neuroscience, Surgery and Trauma, Blizard Institute, Queen Mary University of London, London, UK

**Keywords:** Headache disorders, Chronic migraine, Anamnesis, Diagnosis, Management, Primary care, Support, Red-flags, Referral

## Abstract

**Background:**

Migraine affects 80.8 million people in Western Europe and is the first cause of disability among people between ages 15 and 49 worldwide. Despite being a highly prevalent and disabling condition, migraine remains under-diagnosed and poorly managed.

**Methods:**

An international, online survey was conducted among 201 general practitioners (GPs) from 5 European countries (France, Germany, Italy, Spain and the UK) who are experienced in the management of headache disorders.

**Results:**

The majority of GPs (82%) did not refer patients with chronic migraine (CM) to migraine specialists. Among those patients, the participants estimated that around 55% received preventive medication. Some differences between countries were observed regarding referral rate and prescription of preventive treatment. Most GPs (87%) reported a lack of training or the need to be updated on CM management. Accordingly, 95% of GPs considered that a migraine anamnesis guide could be of use. Overall, more than 95% of GPs favoured the use of a patient diary, a validated diagnostic tool and a validated scale to assess impact of migraine on patients’ daily life. Similarly, 96% of the GPs considered that the inclusion of warning features (red flags) in an anamnesis guide would be useful and 90% favoured inclusion of referral recommendations.

**Conclusions:**

The results from this survey indicate that more education on diagnosis and management of CM is needed in primary care. Better knowledge on the recognition and management of migraine in primary care would improve both prognosis and diagnosis and reduce impact of migraine on patients’ lives, healthcare utilization and societal burden.

**Supplementary Information:**

The online version contains supplementary material available at 10.1186/s12875-021-01402-2.

## Background

Headache disorders and in particular migraine, are one of the most prevalent and disabling diseases worldwide [1, 2]. Migraine is the leading cause of disability among men and women between ages 15 and 49, accounting for 8.2% of total years lived with disability worldwide [1–3]. In Western Europe, 80.8 million people suffer from migraine according to the latest Global Burden of Disease study [1]. Migraine has an enormous impact on patients’ private, social and professional lives and causes substantial societal burden [3–5].

Approximately 2.5% of people with episodic migraine (EM) develop chronic migraine (CM) each year [6], which is significantly more disabling than EM [4, 7]. In CM, attacks become more frequent and their clear periodicity is lost. CM is defined as headache occurring 15 or more days per month for more than 3 months, of which, at least 8 days per month, have features of migraine headache [8]. The prevalence estimates of CM range between 1 and 2% worldwide [6, 9].

Accurate diagnosis of headache disorders can be challenging for non-specialists clinicians [10]. Despite being one of the most common reasons for visits to primary care [11], formal training on recognition and management of headache disorders for general practitioners (GPs) is limited [12]. Diagnosis of the three most prevalent headache disorders -tension type headache (TTH), migraine and medication overuse headache (MOH)- is based on medical history or anamnesis [13, 14]. However, when headache presents, patients and physicians are concerned about possible serious underlying causes [14]. Diagnostic uncertainty favours unnecessary ancillary testing (i.e. imaging), is uncomfortable for the patient, increases healthcare costs and delays timely treatment [14].

Both diagnosis and management of patients with migraine are often suboptimal [15, 16]. The European Headache Federation (EHF) recommends that both EM and CM are diagnosed in primary care, but suggests that CM might require specialist management [13]. Several studies have reported that up to 70% of patients with migraine are not aware of their disease and/or have not been properly diagnosed [15, 17]. Even when accurately diagnosed, patients are often undertreated and not satisfied with their treatment [15, 16, 18, 19]. Failure of acute treatment in patients with EM has been linked to higher risk of medication overuse, which may cause MOH and is a risk factor for CM [20].

The present survey has been conducted among GPs with experience in headache disorders from 5 European countries (France, Germany, Italy, Spain and the UK). The aims of this study were to assess current management of CM in primary care and to evaluate the need by non-migraine specialists for guidance to identify and manage disabling and CM.

## Methods

### Study design, participants and survey

My-LIFE anamnesis project was an international, online survey to GPs who are experienced in management of headache disorders, with regard to their current practice, perceived needs and important topics for migraine anamnesis in primary care.

The questionnaire for the online survey was designed by a pan-European Steering Committee with 7 European experts on migraine management. After a literature search, an initial document that included the most important points for a migraine anamnesis guide was developed. This document was used in six simulated consultations with six different healthcare practitioners (HCP) and six different patients in June 2019, three in Barcelona (Spain) and three in London (UK). Simulated consultations were used to gather insights regarding the need and potential usefulness of a migraine anamnesis tool to guide non-specialists in the identification of CM, and to gain information from neurologists, GPs and patients for the development of the survey questionnaire. The Steering Committee developed the survey questionnaire, which consisted of three parts, with 32 items regarding participants’ clinical experience, current clinical practice with regard to patients with disabling and CM, and perceptions on their need of support for the identification of patients with CM and the important topics for a migraine anamnesis guide (Additional file 1). The questionnaire was written in English and translated into local languages (French, German, Italian, and Spanish). It was administered to 201 GPs fulfilling the inclusion criteria between 14^th^ January 2020 and 28^th^ January 2020 through an online platform that ensured data anonymity and confidentiality.

The participants were randomly selected by a fieldwork company taking into account their geographical location to ensure a similar number of participants from each of the five countries. Experience in the management of headache disorders was defined as: (1) having ≥ 2 years of experience in general practice; (2) seeing ≥ 5 patients with headache disorders per week; (3) usually proceeding with the anamnesis of their patients with headache disorders, and (4) currently having ≥ 1 patient suffering from episodic and/or CM under treatment.

Ethics Committee approval was not applicable in this survey because its objective was to understand chronic migraine patients’ management in the primary care setting according to participants recall. There was no need to collect any type of patient data or information, hence the approval of an Ethics Committee or patient informed consent was not required. All survey participants gave their written consent.

### Data analysis

Number of respondents was expressed as frequencies, percentages, means, standard deviations (SD) and range (min–max). Comparative analyses were carried out with Student’s t-tests, ANOVA or Chi-square tests. Data were analysed with SPSS version 22, and *p *< 0.05 was considered statistically significant. In this study all analyses are simply exploratory so no adjustment for multiple testing was conducted.

## Results

### Simulated consultations

Four simulated consultations were carried out by neurologists, and two by GPs. Neurologists felt that an anamnesis guide to identify and manage CM was not needed for migraine specialists but could be useful for GPs. GPs considered that inclusion of red flags for secondary headaches was helpful. Regarding patients’ perspective, patients felt that the use of a diary was very useful as it helped them to better articulate the migraine experience and its frequency and impact on daily life.

### Online survey

#### Participants’ profile

A total of 201 GPs with experience on the management of headache disorders from France, Germany, Italy, Spain and the UK participated in the online survey. On average, participants had 24 years of clinical practice experience and saw 202 patients per week, of whom 12% suffered from headache disorders, of which 38% were diagnosed of migraine. Among migraine patients 66% presented EM and 34% CM. Detailed information about how identification, diagnosis and follow-up of patients with episodic migraine and CM are conducted by the GPs in this survey is reported separately [21].

#### Management of patients with disabling and CM

**Fig. 1:**
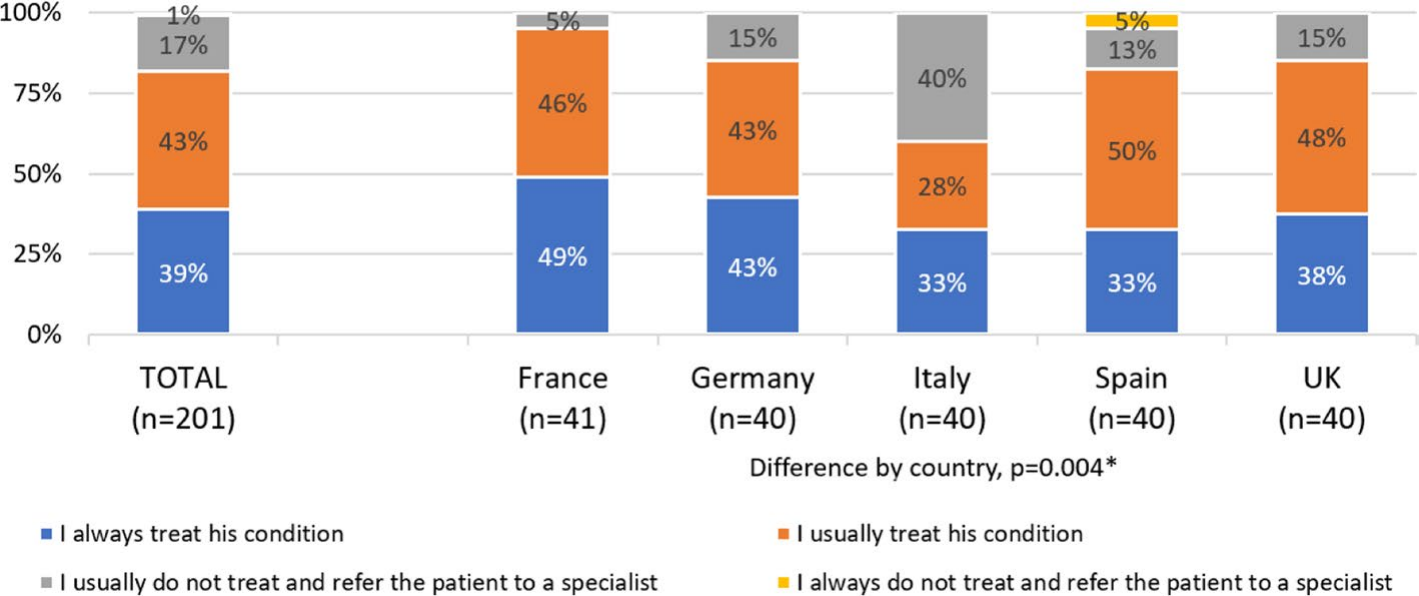
Referral rates of patients with chronic migraine. Legend: *p < 0.05

Up to 82% of GPs continued to manage patients after they were diagnosed with “disabling or chronic migraine” without referring them to migraine specialists, with only 18% of GPs usually or always referring these patients to migraine specialists (Fig. 1). Referral rates differed between countries, ranging from 5% in France to 40% in Italy (*p *= 0.004) (Fig. 1).

Among those GPs who continued with the management of patients with CM without referring them to migraine specialists, 82% treated them with acute medication (analgesics, anti-emetics or specific anti-migraine medication), and 72% with preventive medication, when the frequency or intensity of the migraine episodes required it (Table 1). Of note, the proportion of GPs prescribing acute and preventive treatment differed significantly between countries (Table 1).

**Table 1:** Prescription patterns of GPs who do not refer patients with CM

	**TOTAL** **n = 164**	**France** **n = 39**	**Germany** **n = 34**	**Italy** **n = 24**	**Spain** **n = 33**	**UK** **n = 34**	***p value*** **between countries**
**GPs who prescribe acute medication when needed,** n (%)	134 (82%)	37 (95%)	32 (94%)	19 (79%)	26 (79%)	20 (59%)	*p *= 0.000*
***“% of my patients have been prescribed analgesics or anti-emetics”, ***mean (min, max)	59% (0, 100)	58% (0, 100)	61% (8, 100)	62% (15, 100)	62% (10, 100)	49% (10, 100)	*p *= 0.521
***“% of my patients have been prescribed anti-migraine acute treatment”, ***mean (min, max)	55% (0, 100)	57% (10, 100)	51% (0, 100)	57% (20, 100)	52% (10, 100)	61% (30, 90)	*p *= 0.625
**GPs who prescribe preventive treatment when needed,** n (%)	118 (72%)	27 (69%)	18 (53%)	15 (63%)	26 (79%)	32 (94%)	*p *= 0.003*
***“% of my patients are currently under preventive treatment”*** mean (min, max)	55% (3, 100)	59% (3, 100)	47% (5, 100)	53% (10, 100)	48% (5, 100)	64% (20, 100)	*p *= 0.108

^*^ *p *< 0.05

Regarding type of acute medication, GPs estimated that 59% of their patients with CM had been prescribed with analgesics and/or anti-emetics, and 55% with specific anti-migraine treatment (Table 1). Regarding preventive medication, 72% of the participants prescribed it when needed, and they estimated that 55% of their patients with CM were using preventive treatment (Table 1). Percentages of patients who had received analgesics/anti-emetics, specific symptomatic migraine treatment and/or preventive treatment were similar across countries (Table 1).

Anamnesis guide for the identification and management of CM: need and topics to be included.

Across countries, only 13% of GPs considered that they had received enough education on the management of patients with CM, and 39% responded that there was a lack of training (from 25% in UK to 55% in Italy, without statistically significant differences between countries).

On average, 95% of participants found that an anamnesis guide for patients with headache disorders should be mandatory (23%) or would be helpful (72%) (Table 2). The perceived need for such a guide differed between countries (*p *= 0.009) but was overall high, from 88% in Germany to 100% in Spain (Table 2). Of note, GPs who were currently using an anamnesis guide found it significantly more useful than those who were not currently using it (*p *= 0.017).

**Table 2:** GPs views on need for anamnesis guide and patient diary for chronic migraine

	**TOTAL** **n = 201**	**France** **n = 41**	**Germany** **n = 40**	**Italy** **n = 40**	**Spain** **n = 40**	**UK** **n = 40**	***p value***
**Need for anamnesis guide**							*p *= 0.009*
* Mandatory*	22.9%	22.0%	20.0%	22.5%	42.5%	7.5%	
* Helpful*	71.6%	70.7%	67.5%	75.0%	57.5%	87.5%	
* Needless*	5.5%	7.3%	12.5%	2.5%	0.0%	5.0%	
**Need for a patient diary**							*p *= 0.156
* Mandatory*	31.8%	24.4%	30.0%	32.5%	47.5%	25.0%	
* Helpful*	65.2%	70.7%	62.5%	65.0%	52.5%	75.0%	
* Needless*	3.0%	4.9%	7.5%	2.5%	0.0%	0.0%	
**Inclusion of red flags**							*p *< 0.001*
* Mandatory*	55.2%	51.2%	42.5%	25.0%	72.5%	85.0%	
* Helpful*	40.8%	48.8%	52.5%	62.5%	27.5%	12.5%	
* Needless*	4.0%	0.0%	5.0%	12.5%	0.0%	2.5%	
**Inclusion of referral recommendations**							*p *< 0.001*
* Mandatory*	30.8%	17.1%	17.5%	10.0%	72.5%	37.5%	
* Helpful*	58.7%	73.2%	60.0%	77.5%	25.0%	57.5%	
* Needless*	10.4%	9.7%	22.5%	12.5%	2.5%	5.0%	
**Inclusion of a validated scale to measure impact of migraine on patient’s daily life**							*p *= 0.188
* Mandatory*	28.4%	24.4%	22.5%	20.0%	40.0%	35.0%	
* Helpful*	65.2%	65.9%	65.0%	75.0%	55.0%	65.0%	
* Needless*	6.5%	9.8%	12.5%	5.0%	5.0%	0.0%	
**Inclusion of a validated diagnostic tool**							*p *= 0.003*
* Mandatory*	30.8%	36.6%	22.5%	7.5%	42.5%	45.0%	
* Helpful*	64.2%	61.0%	70.0%	80.0%	55.0%	55.0%	
* Needless*	5.0%	2.4%	7.5%	12.5%	2.5%	0.0%	

^*^ *p *< 0.05

**Fig. 2:**
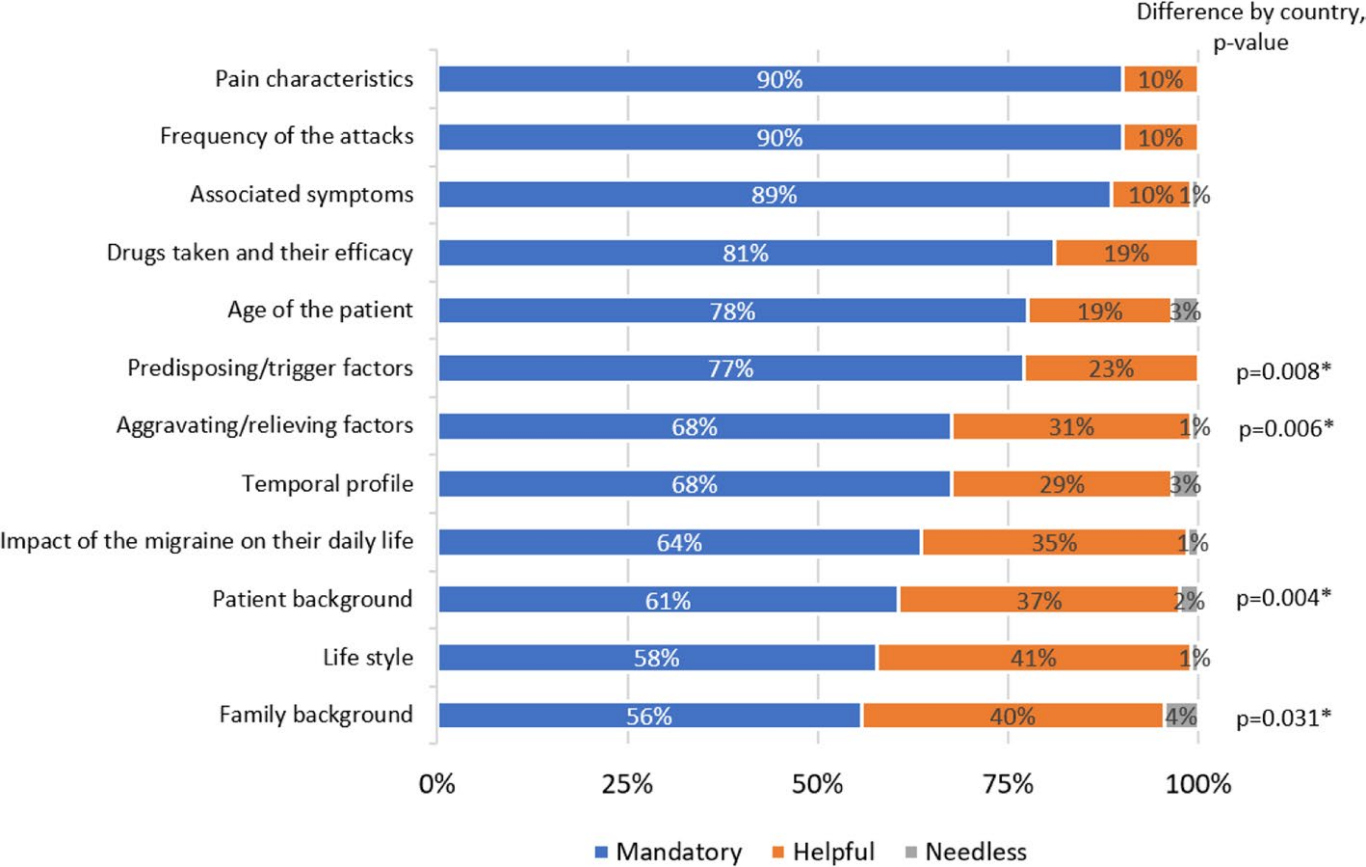
Topics that need to be included in an anamnesis guide for chronic migraine. Legend: *p < 0.05

More than 75% of participants considered mandatory the inclusion of the following topics in an anamnesis guide for migraine: pain characteristics, frequency of attacks, associated symptoms, treatments used and their efficacy, patient’s age and trigger factors (Fig. 2). Some differences were found between countries in judging as mandatory the inclusion of trigger factors (ranging from 60% in Italy to 93% in Spain, *p *= 0.008), aggravating factors (from 48% in Italy to 85% in the UK, *p *= 0.006) and patient background (from 40% in Italy to 88% in Spain, *p *= 0.004) (Fig. 2).

The use of a patient diary was favoured by 97% of participants (considered “mandatory” or “helpful”), with no differences between countries (Table 2). Those GPs who did not normally use a patient diary in their clinical practice found it less important than those who regularly used it (*p *= 0.036).

**Fig. 3:**
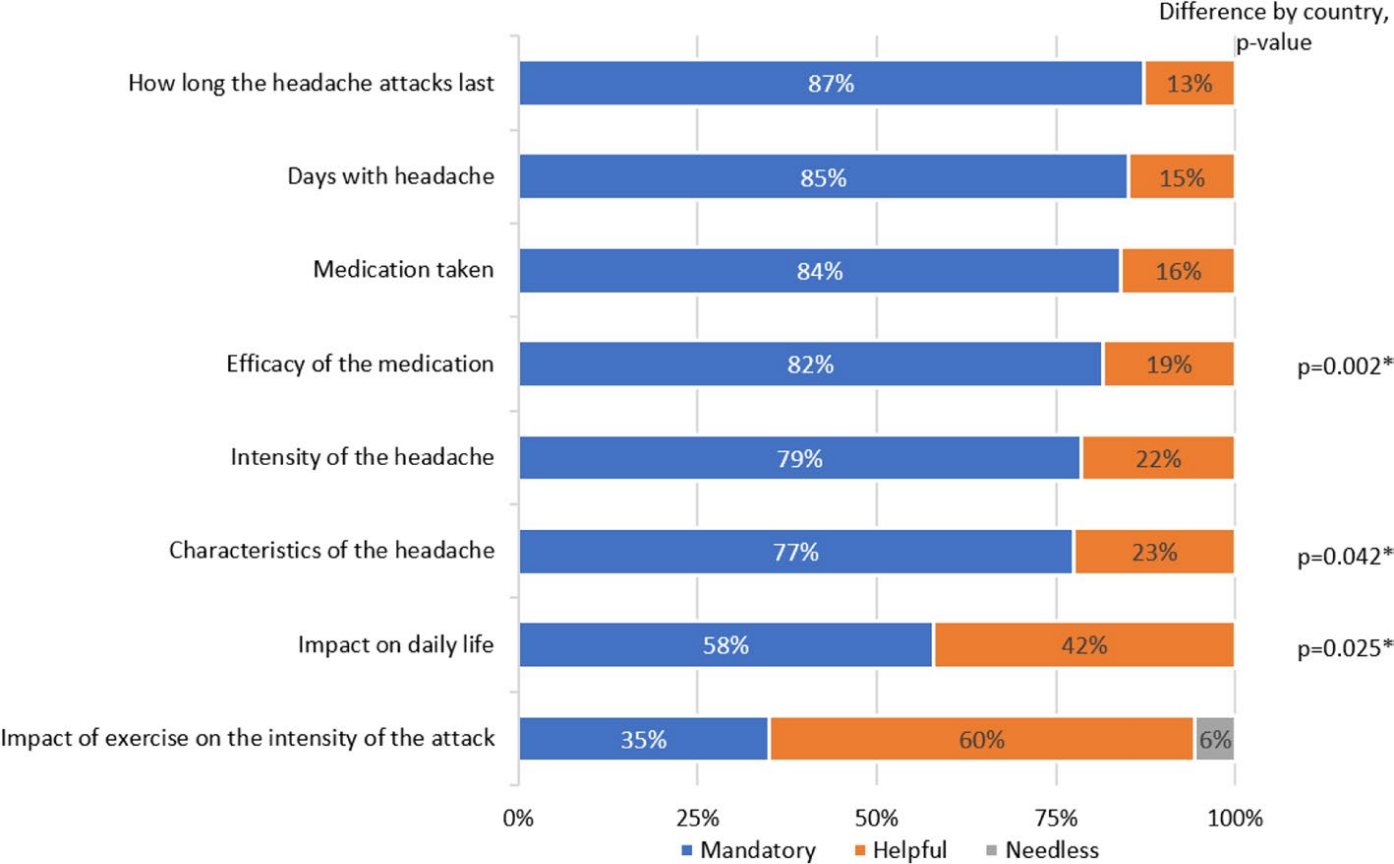
Topics that need to be included in the patient diary. Legend: *p < 0.05

Regarding the topics that should be recorded in a patient diary, more than 75% of participants considered mandatory the inclusion of: headache attack duration, intensity and characteristics, number of headache days, and medications used and their efficacy (Fig. 3). Some differences were seen between countries (Fig. 3).

Regarding the inclusion of red flags/warning features in a migraine anamnesis guide, 96% of GPs found that their inclusion would be beneficial, with differences between countries in judging it mandatory: from 25% in Italy to 85% in the UK (*p *< 0.001) (Table 2).

More than 90% of participants favoured the inclusion of referral recommendations, a validated diagnostic tool and a validated scale to measure the impact of migraine on patients’ daily life (Table 2). Differences were seen between countries as to whether recommendations should be considered mandatory, from 73% of GPs in Spain to only 10% in Italy (*p *< 0.001). Similarly, inclusion of a validated diagnostic tool was considered mandatory by 7.5% of Italian GPs, versus 42% of GPs in Spain and 45% in UK (Table 2).

Lastly, participants had different preferences regarding the format of the guide (checklist or short descriptive list, electronic or paper) (data not shown).

## Discussion

Our results emphasize the need for guidance on the identification and management of patients with CM in family practice across Europe. While only 13% of GPs considered that they had received sufficient training on the management of CM, 82% did not refer patients to migraine specialists when CM was diagnosed, in contrast to guidelines recommendations. Among the GPs who proceeded with the management of patients with CM without referring, 82% prescribed acute treatment and 72% preventive treatment, when needed. Those GPs who did not refer after CM diagnosis estimated that 55% of their patients were currently receiving preventive treatment. As pointed out in the previously published results from this survey, CM treatments prescribed in the primary setting are not in accordance with local and international recommendations [21].

CM is complex and difficult to manage, as it involves not only more frequent migraine but is usually accompanied by different comorbidities and medication overuse [13]. According to European and local current guidelines, CM should be recognised and diagnosed in primary care, but mainly managed by a specialist [13, 22, 23]. Even though the recently updated “Aids to management of headache disorders in primary care” [13] includes a wide variety of tools to support GPs in the identification of CM, in this study, only 18% of GPs considered it necessary to refer their patients to migraine specialists when CM was diagnosed, supporting the need for improved guidance and continuing medical education in the primary care setting.

The assessment of headache disorders patients in the emergency department (ED) setting differs from the assessment in primary care, and a recent work aimed to analyse the different stages of the ED headache management to identify those deficiencies that can be overcome by a fast referral to a headache clinic [24]. The authors found that 75% of patients underwent head computed tomography, 19.3% received a neurological consultation, 43% did not receive any pharmacological treatment and 62.7% still had headache at discharge [24]. Furthermore, the concordance analysis showed a low agreement for the diagnosis between triage nurse, ED physicians and headache centre [24]. All these issues could be avoided if those patients had been correctly diagnosed in the primary care and referred to headache specialists when needed [24]. In this sense, the Spanish Society of Neurology’s Headache Study Group (GECSEN) has recently published a series of recommendations that aim to help ED and primary care physicians in the management of patients with headache and optimize their referral to specialists [25, 26]. Moreover, prescription of some preventive treatments for CM is restricted to migraine specialist care in most of the European countries [13]. The low referral rate reported by GPs in our study suggests a high percentage of under-treatment of patients with CM.

Several studies have shown that patients with CM are very commonly undertreated [16, 19, 27]. Suboptimal treatment is also observed in our study, with 55% of the patients with CM treated by GPs currently receiving preventive treatment. Preventive treatment is recommended in patients with uncontrolled migraine despite the use of acute medication, or who experience frequent (4 or more) attacks per month [22]. Optimization of migraine treatment is important for prognosis and patients’ quality of life. Acute treatment failure cause more frequent attacks, which contribute to the risk of CM [28]. Transformation of a common episodic migraine into a chronic headache disorders is also associated with medication overuse [13], and a systematic review of case-controlled and longitudinal studies identified headache days frequency, depression and medication overuse/high-frequency use as risks factors for developing CM [20].

The differences observed among countries could be related to differences between healthcare systems dynamics, patient’s journey and availability of information on headache disorders management that is targeted to GPs. In the UK, the British Association for the Study of Headache (BASH) guidelines for the diagnosis and management of headache disorders include comprehensive information on CM management and are directed to all HCP [22]. In this sense, more GPs in the UK prescribed preventive treatment to patients with CM (94% in the UK compared to an average of 72% across countries) and less prescribed acute treatment (59% vs. 82%, respectively).

Despite being one of the most prevalent and disabling group of diseases, formal training on Headache Medicine is very limited [12, 29] and some knowledge gaps on management of headache disorders such as CM have been repeatedly reported in both family practice and specialist settings [15, 29–32]. In agreement with this, only 13% of GPs considered that they had received enough education about management of CM.

In our project, 95% of GPs considered helpful or mandatory the use of an anamnesis guide for patients with headache disorders. Diagnosis and management of headache disorders can be challenging for non-expert physicians [10]. A comprehensive tool to help diagnose headache disorders, in particular chronic headache disorders, has been lacking [10]. Several tools to help in the identification of migraine and CM (e.g., ID-Migraine [33], ID-CM [34]) are available; however, the majority of them are self-administered questionnaires that do not include warning features, referral recommendations or tools to assess impact of migraine on patients’ lives [10]. In contrast, more than 90% of GPs in this survey considered helpful or mandatory the inclusion of these features in an anamnesis guide. The inclusion of red flags in a diagnostic tool is of high importance to help the differential diagnosis of any serious secondary headache disorders that requires specific treatment and/or referral [35]. In agreement with that, the majority of GPs in the survey considered useful their inclusion in an anamnesis guide.

Diagnosis of the most common headache disorders should be accurately done in primary care [13, 36]. A comprehensive tool that provides guidance for migraine anamnesis would reduce unnecessary referral of patients with episodic migraine and provide better care of patients with CM by speeding up referral and timely access to preventive treatment. The recent update of the “Aids to management of headache disorders in primary care” [13], contains a number of tools that have been requested by the GPs in this survey, such as a diagnosis guide of headache disorders including warning features, aids for management of CM, referral recommendations, and a headache diary and calendar.

This project has some limitations. It is based on a survey and therefore relies on respondents’ recall. Taking into consideration the inclusion criteria defined to participate in the survey, even if not headache specialists, the GPs’ experience of CM might have influenced the results and can limit their extrapolation to all primary care physicians. We did not include extensive medical information of the patients treated by GPs, and thus we could not evaluate severity of migraine or specific type of treatment received. Lastly, inclusion criteria to participate in the survey could create a certain bias in the results, as participants had some clinical experience with headache disorders. This may suggest that under-treatment of patients with CM in primary care could be higher than reported here.

## Conclusions

Our results suggest that GPs would benefit from greater of application of management guidelines and continuing medical education on diagnosis and management of headache disorders, specifically CM. Better identification of patients with CM would ensure their better management, and optimal referral to migraine specialists when needed improving patient access to more effective preventive treatments.

The vast majority of GPs considered of great importance the use of an anamnesis guide for CM. Most of the features that GPs considered helpful or mandatory in our survey (i.e., a headache diary, red flags, referral recommendations, and a validated scale to assess impact of migraine on patients’ daily life) are included in the 2019 update of the “Aids to management of headache disorders in primary care” [13].

## Supplementary Information

**Additional file 1.** Online questionnaire. 32-item questionnaire run online among GPs.

## Data Availability

The datasets used and/or analysed during the current study are available from the corresponding author on reasonable request.

## Abbreviations

BASH: British Association for the Study of Headache
CM: Chronic migraine
EM: Episodic migraine
ED: Emergency Department
EHF: European Headache Federation
GP: General practitioner
HCP: Healthcare practitioner
MOH: Medication-overuse headache
TTH: Tension-type headache
