# Supplementary material for: Need of guidance in disabling and chronic migraine identification in the primary care setting, results from the european MyLife anamnesis survey
Source: BMC Fam Pract. 2021 Mar 20;22:54. doi: 10.1186/s12875-021-01402-2 (PMC7980340; doi:10.1186/s12875-021-01402-2)
Supplement: Supplementary file 1 — Additional file 1. Online questionnaire. 32-item questionnaire run online among GPs. [file 12875_2021_1402_MOESM1_ESM.docx]

**Additional file 1**

# Online questionnaire

# Introduction

The objective of this questionnaire is to gather your opinion on the need of a Migraine Anamnesis Guide to support you in the identification of patients with chronic and disabling migraine, its content and its usefulness for routine clinical practice.

The questionnaire has 3 sections:

1. Background
2. Current clinical practice in chronic and disabling migraine identification and treatment
3. Evaluation of a Migraine Anamnesis Guide

There is no correct nor incorrect answer, what we would like to know is your current clinical practice and your opinion on the topics that needs to be approached in a Migraine Anamnesis Guide.

Please, answer all questions the more precisely and sincerely you can.

Many thanks for your collaboration.

***My-LIFE Anamnesis guide project team.***

Please, click here to start the questionnaire

### Background/Screener

1. Medical speciality:
   1. General practitioner
   2. Neurologist
   3. Internal medicine
   4. Psychiatrist
   5. Other: please specify:

(* only “General practitioners” can participate in this survey, the participation of other medical specialities will be cancelled)

2. For how long have you been working as a General practitioner?

______ years

(* if < 2 years the participation of the panelist will be cancelled)

3. How many patients do you see in 1 week?

______ patients

4. Among these patients, how many of them are suffering from headache disorders?

______ patients

(* if < 5 the participation of the panelist will be cancelled)

5. Please classify your patients suffering from headache disorders according to the type of headache disorder listed below:

| Migraine | % |
| --- | --- |
| Tension-type headache | % |
| Cluster headache | % |
| Medication-overuse headache | % |
| Others | % |

(*the total can sum > than 100%)

6. Do you usually proceed with the anamnesis of your patients with headache disorders?

- Yes
- No, when I suspect a headache disorder, I refer the patient to a specialist.

(* if “No” the participation of the panelist will be cancelled)

7. Considering the following definitions form *Steiner TJ et al.* [*J Headache Pain.*](https://www.ncbi.nlm.nih.gov/pubmed?term=(steiner%5BAuthor%20-%20First%5D)%20AND%20Aids%20to%20management%20of%20headache%20disorders%20in%20primary%20care%20(2nd%20edition))*2019 May 21;20(1):57*:

Episodic migraine: recurrent attack-like episodes, lasting from 4h to 3 days, frequency often 1-2/month but variable from 1/year to 2/week or more; freedom from symptoms between attacks.

Chronic migraine: episodic lost: headache on ≥ 15 days/month, having migrainous features on ≥8 days/month.

7a. How many of your patients are suffering from episodic migraine?

______ patients

7b. How many of your patients are suffering from chronic migraine?

______ patients

(* if “7a” +”7b” are equal to “0” the participation of the panelist will be cancelled)

8. Who did the diagnosis of episodic migraine of those patients?

- I did it, for the majority of them.
- It was done by a specialist, for the majority of them.

9. Who did the diagnosis of chronic migraine of those patients?

- I did I, for the majority of them.
- It was done by a specialist, for the majority of them.

### Current clinical practice in disabling or chronic migraine identification and treatment

10. During the anamnesis of a patient with a headache disorder, do you usually rule out tension type headache?

- Always
- Sometimes
- Never

(* only 1 answer is possible)

11. During the anamnesis of a patient with a headache disorder, do you usually rule out medication over use?

- Always
- Sometimes
- Never

(* only 1 answer is possible)

12. When facing patient with disabling or chronic migraine, when do you use the following tools?

|  | 1^st^ visit | Follow-up visit |
| --- | --- | --- |
| Clinical interview | □ | □ |
| An Anamnesis Guide to diagnose chronic migraine | □ | □ |
| A Patient Diary | □ | □ |
| Imaging techniques (when possible) | □ | □ |
| A validated scale to assess the impact of migraine | □ | □ |
| A validated migraine screening tool | □ | □ |
| Others. Please specify | _____ | _______ |
| None of the above mentioned | □ | □ |

(*more than 1 answer per row & column are possible, if “none of the above mentioned” no other answer in the same column is allowed)

(13. If Q12 “Anamnesis Guide” mentioned:)

13a. What kind of Anamnesis Guide are you currently using?

- A guide/document that I’ve developed myself.
- An Anamnesis Guide developed by the centre where I work.
- An Anamnesis Guide validated and published

(* only 1 answer is possible)

13b. Which of the following aspects are included in the Anamnesis Guide that you are currently using? (Mark all that apply)

- Age of the patient
- Patient background/health history
- Family background/health history
- Temporal pattern of the attacks
- Frequency of the attacks
- Life style
- Pain characteristics of the attacks (intensity, location, duration, …)
- Associated symptoms of the attacks (photophobia, phonophobia, nausea, …)
- Trigger/aggravating factors
- Activity of the patient during the attack
- Medication used, frequency, effectiveness
- Patient perception of the efficacy of the medication used
- Impact of the migraine on their daily life (Pain restricting activity)
- Others: Specify: ______

(* more than 1 answer is possible)

13c. Does the Anamnesis Guide that you are currently using, includes warning features or “red flags”?

- Yes
- No

(* only 1 answer is possible)

(14. If Q12 “Anamnesis Guide” is NOT mentioned)

14. What topics do you usually address during the anamnesis of patients suffering from headache disorders? (Mark all that apply)

- Age of the patient
- Patient background/health history
- Family background/health history
- Temporal pattern of the attacks (When did the attacks started?)
- Frequency of the attacks (How often does the patient suffer from attacks?)
- Life style
- Pain characteristics of the attacks (intensity, location, duration, …)
- Associated symptoms of the attacks (photophobia, phonophobia, nausea, …)
- Trigger/aggravating factors
- Activity of the patient during the attack
- Medication used, frequency, effectiveness
- Patient perception of the efficacy of the medication used
- Impact of the migraine on their daily life (Pain restricting activity)
- Others. Specify: __________________________

(* more than 1 answer is possible)

(15. If Q12 “Patient Diary” mentioned:)

15a. What kind of Patient Diary do you recommend to your patients?

- I ask the patient to record some items in a notebook
- I use a standard Patient Diary we have where I work
- I use a validated and/or published Patient Diary

(* only 1 answer is possible)

15b. What are the main items recorded in the Patient Diary you are currently using? (Mark all that apply)

- Days with headache
- How long the headache lasts
- Characteristics of the headache (location, type of pain, …)
- Intensity of the headache
- Medication taken
- Impact on daily life
- If the attack is made worse when doing exercise
- Others. Specify: ___________________

(* more than 1 answer is possible)

(16. If Q12 “imaging” mentioned:)

16. When do you proceed with imaging techniques?

- Always
- Sometimes, to rule out secondary headaches
- Sometimes, to reassure the patient
- Never

(* if “always” or “never” no other answers can be marked. If “sometimes” both answers are possible.)

(17. If Q12 “validated scale or migraine screening tool” mentioned:)

17. Please, indicate which of the following scales do you use and when:

|  | 1^st^ visit | Follow-up visit |
| --- | --- | --- |
| ID-Migraine  Identify migraine | □ | □ |
| ID-Chronic migraine  Identify chronic migraine | □ | □ |
| HALT-90 or HALT-30  Headache attributed lost time | □ | □ |
| HIT-6  Headache impact test | □ | □ |
| HURT  Headache under response to treatment | □ | □ |
| MAT Migraine Assessment Tool | □ | □ |
| MIDAS  Migraine disability assessment | □ | □ |
| Other: Please specify | ______ | ______ |
| None | □ | □ |

(*more than 1 answer per row & column are possible, if “none” no other answer is allowed)

18. Once the patient is diagnosed with “disabling or chronic migraine”, which of the following sentences best fits to your daily practice:

- I always treat his condition.
- I usually treat his condition.
- I usually do not treat and refer the patient to a specialist.
- I always do not treat and refer the patient to a specialist.

(* only 1 answer is possible)

(19. If Q18 “I treat his condition (always or usually)”:)

19. What kind of treatments do you prescribe to your patients suffering from disabling or chronic migraine?

- When needed, I treat their condition with acute medication like analgesics, antiemetics or specific anti-migraine drugs.
  - _____ % of my patients suffering from disabling or chronic migraine have been prescribed any analgesic or antiemetic drug.
  - _____ % of my patients suffering from disabling or chronic migraine have been prescribed a specific anti-migraine acute treatment.
- When needed, I treat their condition with migraine prophylactic treatments.
  - _____ % of my patients suffering from disabling or chronic migraine are under prophylactic treatment.

(*both answers are possible)

(20. If Q18 “I refer to a specialist (always or usually)”:)

20a. Why do you refer your patients with disabling or chronic migraine to a specialist? (Tick all that apply)

- To confirm the diagnosis
- To rule out any secondary headache
- To give them access to prophylactic treatments
- Others. Please specify: ______

(*all answers are possible)

20b. Even if you refer your patients with disabling or chronic migraine to a specialist. Are you in charge of their follow-up?

- Yes, I’m in charge of the follow-up of all my patients diagnosed with disabling or chronic migraine
- Yes, I’m in charge of the follow-up of some of my patients diagnosed with disabling or chronic migraine
- No, the follow-up is done by the specialist

(* only 1 answer is possible)

(21. If Q19: “I treat his condition with acute medication”)

21a. How do you evaluate the efficacy of the prescribed acute treatment? (Tick all that apply)

- I ask for the patient perception
- I ask the patient to fill in a diary in order to evaluate the frequency of the migraine attacks
- I use a validated scale to evaluate the impact of migraine on the patient daily life

(*all answers are possible)

21b. How often do you, on average, evaluate the efficacy of the prescribed acute treatment?

- On a weekly basis
- On a monthly basis
- On a quarterly basis
- On a semester basis
- On a yearly basis
- When the patient comes back and complains again about his headache disorder

(* only 1 answer is possible)

(22. If Q19: “I treat his condition with migraine prophylactic treatments”)

22a. How do you evaluate the efficacy of the prescribed prophylactic treatment? (Tick all that apply)

- I ask for the patient perception
- I ask the patient to fill in a diary in order to evaluate the frequency of the migraine attacks
- I use a validated scale to evaluate the impact of migraine on the patient daily life

(*all answers are possible)

22b. How often do you, on average, evaluate the efficacy of the prescribed prophylactic treatment?

- On a weekly basis
- On a monthly basis
- On a quarterly basis
- On a semester basis
- On a yearly basis
- When the patient comes back and complains again about his headache disorder

(* only 1 answer is possible)

(23. If Q20b: “Yes, I’m in charge of the follow-up of all/some of my patients”)

23a. How do you evaluate the efficacy of the treatment prescribed by the specialist? (Tick all that apply)

- I ask for the patient perception
- I ask the patient to fill in a diary in order to evaluate the frequency of the migraine attacks
- I use a validated scale to evaluate the impact of migraine on the patient daily life

(*all answers are possible)

23b. How often do you, on average, evaluate the efficacy of the treatment prescribed by the specialist?

- On a weekly basis
- On a monthly basis
- On a quarterly basis
- On a semester basis
- On a yearly basis
- When the patient comes back and complains again about his headache disorder

(* only 1 answer is possible)

(If Q19a: “I treat his condition with acute medication” or if Q19b: “I treat his condition with migraine prophylactic treatments” or if Q20b: “Yes, I’m in charge of the follow-up of all/some of my patients”)

24. When do you consider that your patient with disabling and/or chronic migraine needs to be referred to a headache specialist? (Tick all that apply)

- Diagnostic uncertainty
- When Migraine is highly disabling
- When Migraine with aura is suspected
- When Chronic Migraine is suspected
- When the patient is not answering to the acute medication prescribed
- When the patient is not answering to the prophylactic treatment prescribed
- When quality of life is negatively affected by the migraine
- When the frequency of migraine attacks increases
- When the intensity of migraine attacks increases
- Others. Specify:________________________

(*all answers are possible)

25. Do you consider that during your education as General Practitioner you’ve been adequately trained on how to manage disabling and chronic migraine patients?

- Yes, I’ve received enough training
- Yes, I’ve received enough training however some update would be of interest
- No, I think there is some lacks in this regard.

(* only 1 answer is possible)

### Definition of a Migraine Anamnesis Guide

In order to gather your opinion on a Migraine Anamnesis Guide content and its usefulness for your routine clinical practice, we would like that you answer the following questions considering that the patient comes to your office for the 1^st^ time complaining about multiple attacks of headache, and that you suspect he/she is suffering from a disabling or chronic migraine.

26. Do you consider that a document to guide you during the anamnesis of a patient with headache disorder and support you when referral is needed, would be…

- Mandatory
- Helpful
- Needless

(* only 1 answer is possible)

27. What are the topics/questions you consider that need to be included in a Migraine Anamnesis Guide:

|  | Mandatory | Helpful | Needless |
| --- | --- | --- | --- |
| Age of the patient | □ | □ | □ |
| Patient background | □ | □ | □ |
| Family background | □ | □ | □ |
| Temporal profile | □ | □ | □ |
| Frequency of the attacks | □ | □ | □ |
| Life style | □ | □ | □ |
| Pain characteristics (headache onset, intensity, location, duration, quality, …) | □ | □ | □ |
| Associated symptoms (nausea, photophobia, phonophobia, osmophobia,) | □ | □ | □ |
| Predisposing/trigger factors | □ | □ | □ |
| Aggravating/relieving factors | □ | □ | □ |
| Impact of the migraine on their daily life (Pain restricting activity) | □ | □ | □ |
| Drugs taken during the attacks and their efficacy | □ | □ | □ |
| The order of the topics approached during the anamnesis needs to be defined in a Migraine Anamnesis Guide | □ | □ | □ |
| Others. Specify | _____ | | |

(*only 1 answer per row)

28. Do you consider that a Patient Diary kept over a few weeks to support you during the diagnosis of disabling or chronic migraine would be…?

- Mandatory
- Helpful
- Needless

(* only 1 answer is possible)

(29. if Q28: “mandatory” or “helpful”)

29. In your opinion, what are the key topics that need to be recorded in a Patient Diary?

|  | Mandatory | Helpful | Needless |
| --- | --- | --- | --- |
| Days with headache | □ | □ | □ |
| How long the headache attacks last | □ | □ | □ |
| Characteristics of the headache | □ | □ | □ |
| Intensity of the headache | □ | □ | □ |
| Medication taken | □ | □ | □ |
| Efficacy of the medication | □ | □ | □ |
| Impact on daily life | □ | □ | □ |
| Impact of exercise on the intensity of the attack | □ | □ | □ |
| Others. Specify | ____ | | |

(*only 1 answer per row)

30. Do you consider that including the following topics in a Migraine Anamnesis Guide to guide during the identification of disabling or chronic migraine would be….

|  | Mandatory | Helpful | Needless |
| --- | --- | --- | --- |
| Warning features / “red flags” | □ | □ | □ |
| Validated diagnostic tool | □ | □ | □ |
| Validated scale to measure the impact of migraine on the patient daily life | □ | □ | □ |
| Referral recommendations | □ | □ | □ |
| Others. Specify | ____ | | |

(* only 1 answer per row)

31. When considering a Migraine Anamnesis Guide to support you in the diagnosis of disabling or chronic migraine. What format would be the most useful for your daily practice?

- Extensive and detailed document, including the questions to ask, as well as the tools to use, and the recommendations on how to proceed
- A short descriptive document with recommendations on how to proceed.
- Check list with the key topics to approach during the anamnesis.

(* only 1 answer is possible)

32. What kind of support would be more useful in your daily practice for a Migraine Anamnesis Guide to support you in the diagnosis of disabling or chronic migraine.

- Printed document
- Downloadable document
- On line document
- Mobile phone App

(* only 1 answer is possible)

You have reached the end of the questionnaire. Many thanks for your valuable collaboration.

***My-LIFE Anamnesis Guide project team***
